# Prognostic value of HELT-E_2_S_2_ score among patients undergoing percutaneous coronary intervention: sub-analysis of the SHINANO 5-year registry

**DOI:** 10.1007/s12928-025-01123-8

**Published:** 2025-03-29

**Authors:** Daisuke Sunohara, Tatsuya Saigusa, Yasushi Ueki, Masatoshi Minamisawa, Tadashi Itagaki, Yoshiteru Okina, Kiu Tanaka, Hidetomo Nomi, Tamon Kato, Soichiro Ebisawa, Takashi Miura, Koichiro Kuwahara

**Affiliations:** 1 Department of Cardiovascular Medicine, Shinshu University School of Medicine, Asahi 3-1-1, Matsumoto, Nagano, 390-8621 Japan; 2 Department of Cardiology, Nagano Municipal Hospital, Nagano, Japan

**Keywords:** Clinical outcomes, Coronary artery disease, Percutaneous coronary syndrome

## Abstract

The HELT-E_2_S_2_ score has recently been proposed as a new risk stratification tool for ischemic stroke in Japanese patients with atrial fibrillation (AF). We aimed to evaluate the prognostic value of HELT-E_2_S_2_ score for cardiovascular events among patients undergoing percutaneous coronary intervention (PCI). Between August 2012 and July 2013, patients undergoing PCI enrolled in the SHINANO 5-year registry, a prospective, observational multicenter registry, were analyzed for the current study. Primary endpoint was major adverse cardiovascular events (MACE), a composite of all-cause death, myocardial infarction, and ischemic stroke at 5 years. Key secondary endpoint was bleeding events at 5 years. A total of 1608 patients were divided into the low HELT-E_2_S_2_ score group (< 2, n = 915) and high HELT-E_2_S_2_ score group (≥ 2, n = 693). Major criteria of the HELT-E_2_S_2_ score were hypertension (74.7%), elderly age 75–84 years (31.3%), and AF (11.1%). Kaplan–Meier analysis revealed that the high HELT-E_2_S_2_ score group had a significantly higher incidence of MACE (33.4% vs. 14.8%, P < 0.001) and bleeding events (12.7% vs. 5.3%, P < 0.001) compared with the low HELT-E_2_S_2_ score group. Multivariable analysis demonstrated that the HELT-E_2_S_2_ score remained significantly associated with MACE (hazard ratio: 1.73, 95% confidence interval: 1.11–2.69; P = 0.015). The HELT-E_2_S_2_ score appears a useful tool for predicting future cardiovascular events in CAD patients undergoing PCI.

**Graphical abstract:**

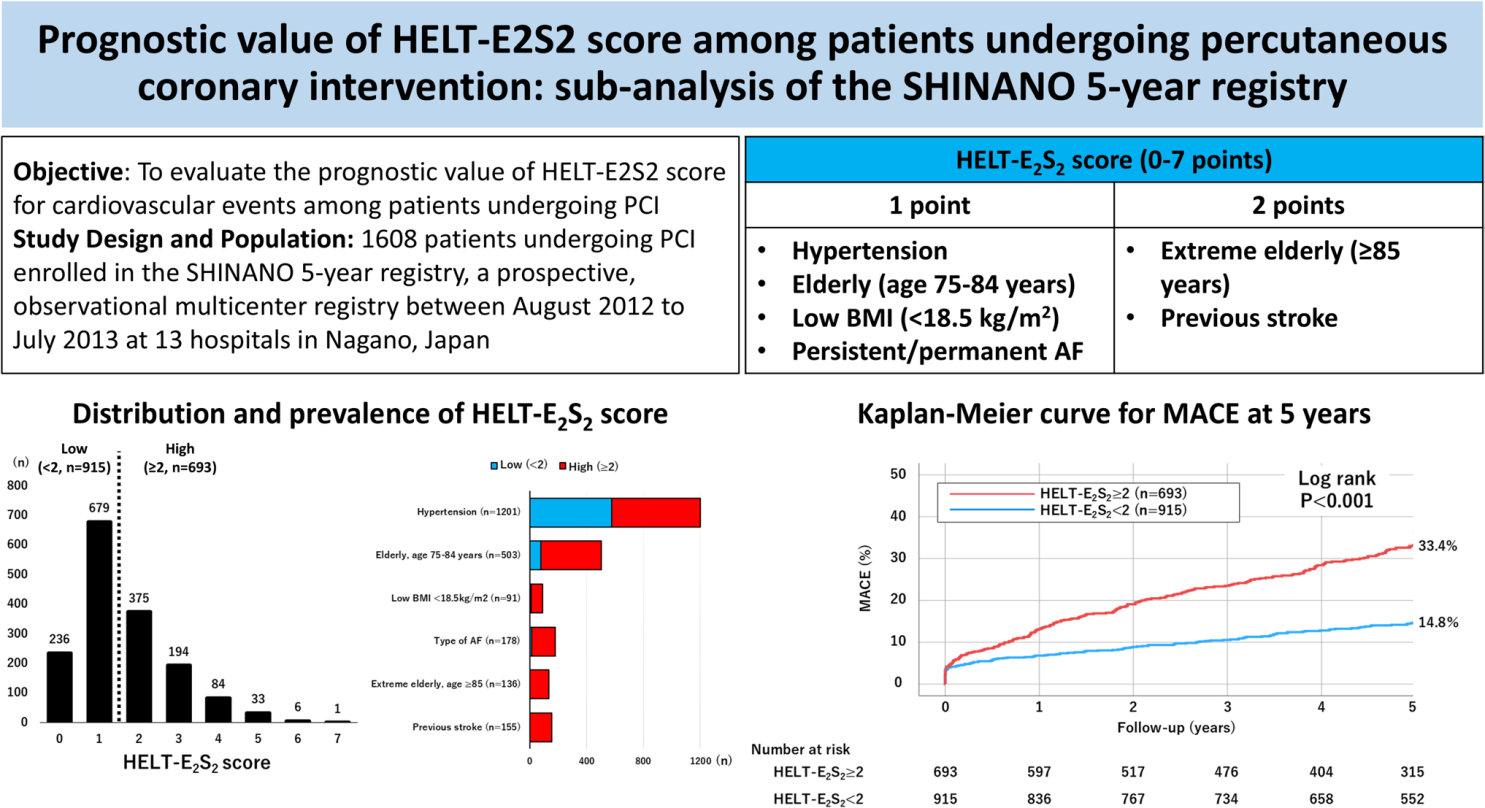

**Supplementary Information:**

The online version contains supplementary material available at 10.1007/s12928-025-01123-8.

## Introduction

CHADS_2_ and CHA_2_DS_2_-VASc scores have been applied to predict the risk of embolization and thus guide the initiation of anticoagulation therapy in patients with atrial fibrillation (AF) [1, 2]. Given the limited generalizability of these scores to Japanese population, the HELT-E_2_S_2_ score was developed using large Japanese registry data for the prediction of ischemic embolization in Japanese patients with AF [3]. The HELT-E_2_S_2_ score incorporates the following readily available clinical characteristics including history of hypertension, elderly (defined as age 75–84 years), low body mass index (BMI) (defined as < 18.5 kg/m^2^), type of AF (persistent or permanent), extreme elderly (defined as age ≥ 85 years), and history of stroke. The HELT-E_2_S_2_ score ≥ 2 was considered to be at an increased risk for ischemic stroke. The HELT-E_2_S_2_ score has been shown to be as useful as CHADS_2_ and CHA_2_DS_2_-VASc scores in predicting the incidence of ischemic stroke among Japanese AF patients [4].

Accurate risk stratification is clinically relevant for patient-centered care and optimal management. Previous studies have demonstrated that the CHADS_2_ and CHA_2_DS_2_-VASc scores were associated with cardiovascular outcomes in patients undergoing percutaneous coronary intervention (PCI) [5–7]; however, to date, the prognostic ability of HELT-E_2_S_2_ score has not been elucidated. Therefore, we aimed to evaluate the utility of HELT-E_2_S_2_ score for predicting cardiovascular events in patients with coronary artery disease (CAD) undergoing PCI.

## Methods

### Patients and study design

This was a post-hoc analysis of the Shinshu prospective multicenter analysis for patients with CAD undergoing PCI (SHINANO) 5-year registry, a prospective, multicenter, observational registry including patients undergoing PCI between August 2012 to July 2013 at 13 hospitals in Nagano, Japan [8]. For the current sub-study, patients in whom the HELT-E_2_S_2_ score could not be ascertained were excluded. The study protocol was developed in accordance with the Declaration of Helsinki and was approved by the ethics committee of each participating hospital.

### HELT-E_2_S_2_ score

In the current study, the presence of AF (irrespective of AF type) was applied due to nonavailability of the type of AF. The HELT-E_2_S_2_ score was calculated by adding 1 point for hypertension, elderly (aged 75–84 years), low BMI < 18.5 kg/m^2^, and AF, and 2 points for extreme elderly (≥ 85 years) and previous stroke, with a range of the score from 0 to 7 [3].

### Endpoints

The primary endpoint in the current study was major adverse cardiovascular events (MACE), defined as a composite of all-cause death, non-fatal myocardial infarction (MI), and non-fatal ischemic stroke at 5 years. The secondary endpoints were each component of MACE and bleeding events at 5 years. Bleeding events were defined as Bleeding Academic Research Consortium (BARC) 2, 3 or 5 bleeding [9]. These outcomes were ascertained through medical records and follow-up questionnaires sent to patients’ primary physician.

### Statistical analysis

Continuous variables were expressed as mean ± standard deviation or medians with interquartile range (IQR) and were compared using the student’s t test or Mann–Whitney test as appropriate. Categorical variables were expressed as frequency (percentages) and were compared using the Fisher’s exact test or the Chi square test. Poisson models were used to estimate incidence rates (100 patient-years). The cumulative incidences were estimated based on the Kaplan–Meier method, and differences were assessed using the log-rank test. A multivariable Cox proportional hazards regression analysis was performed to test the prognostic significance of HELT-E_2_S_2_ score for study endpoints, and adjusted hazard ratios (HR) and 95% confidence intervals (CI) were calculated. The HELT-E_2_S_2_ score was adjusted by clinically important variables reported by previous studies [10]. For MACE and each component, male, dialysis, history of heart failure, diabetes, lower extremity artery disease (LEAD), history of MI, acute coronary syndrome (ACS), left ventricular ejection fraction less than 40%, hemoglobin, estimated glomerular filtration rate (eGFR), and statin use at discharge; and for BARC 2, 3 or 5 bleeding, male, dialysis, history of heart failure, LEAD, ACS, hemoglobin, eGFR, oral anticoagulant (OAC) use at discharge were entered into a multivariate model [11]. Receiver-operating characteristics (ROC) curve analysis was used to evaluate the discriminatory capacity of each parameter in predicting MACE, which were assessed by the C-statistics. Receiver-operating characteristics (ROC) curve analysis was used to evaluate the discriminatory capacity of each parameter, which were assessed by the C-statistics. The C-statistics for MACE and ischemic stroke were compared among the HELT-E_2_S_2_ score, CHADS_2_ score, and CREDO-Kyoto thrombotic risk score [12]. Analysis items with P < 0.05 were considered statistically significant. All analyses were performed with SPSS version 29.0 software (SPSS Inc., Chicago, Ill, USA) and STATA version 18.0 (Stata Corp, College Station, TX, USA).

## Results

### Baseline characteristics

**Fig. 1:**
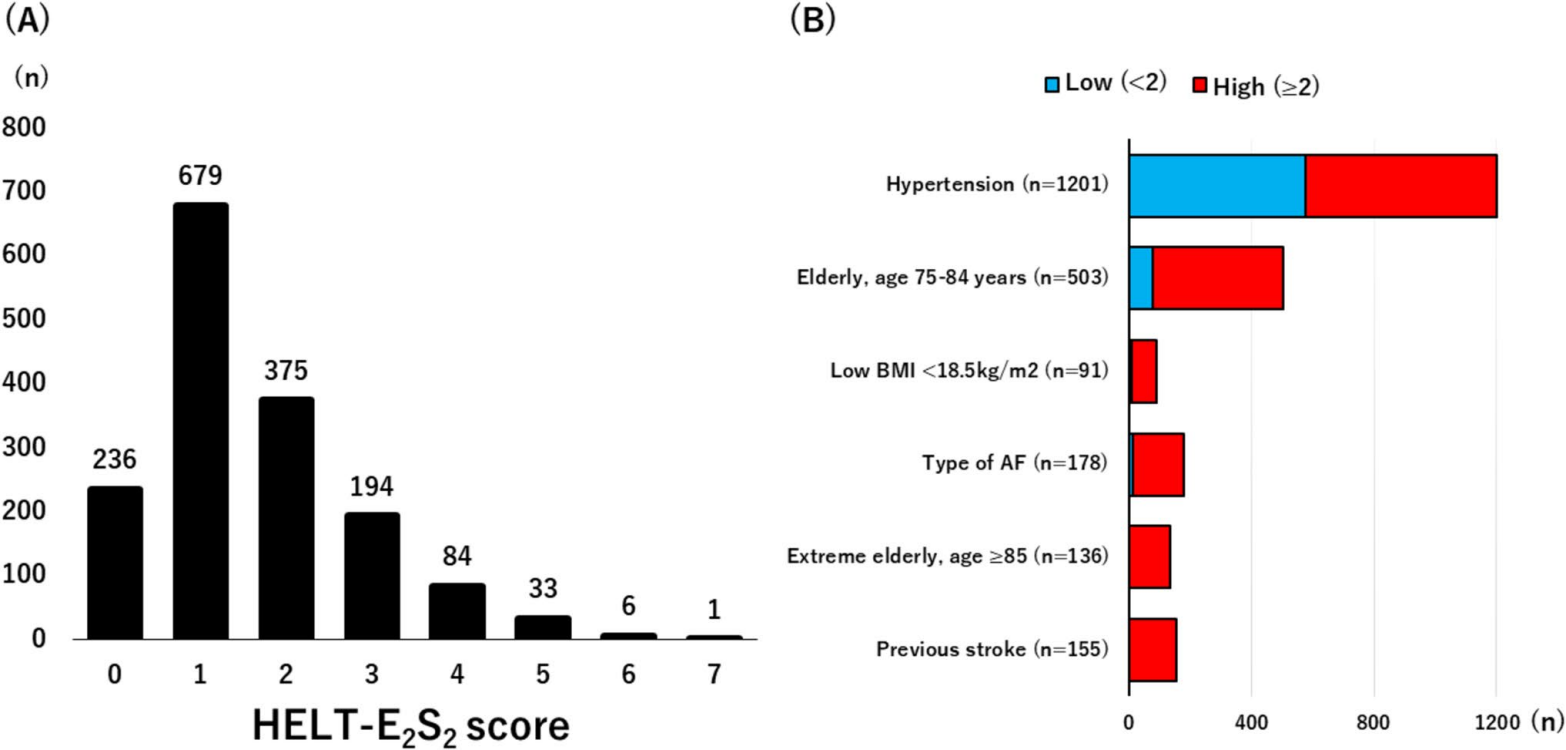
Distribution (**A**) and prevalence (**B**) of HELT-E_2_S_2_ score. *AF* atrial fibrillation, *BMI* body mass index

Of 1665 consecutive patients undergoing PCI, after excluding 57 patients not available for BMI, 1608 patients were analyzed for the current study. Patients were divided into two groups according to the median value of the HELT-E_2_S_2_ score (low HELT-E_2_S_2_ score [< 2, n = 915] and high HELT-E_2_S_2_ score [≥ 2, n = 693]) (Fig. 1A). Major criteria of the HELT-E_2_S_2_ score were hypertension (74.7%), elderly age 75–84 years (31.3%), and type of AF (11.1%) (Fig. 1B). Low BMI, extreme elderly age ≥ 85, and previous stroke frequently overlapped with other criteria as illustrated in Supplementary Fig. 1.

Clinical and procedural characteristics are summarized in Tables 1 and 2. Patients in the high HELT-E_2_S_2_ score group were older and had more atherosclerotic risk factors, comorbidities, chronic coronary syndrome as an indication for PCI, and higher CHADS_2_ and CHA_2_DS_2_-VASc scores compared with those in the low HELT-E_2_S_2_ score group. There were no differences in almost all procedural characteristics between the two groups, except for the use of new-generation drug eluting stent (DES) and mean stent diameter.

**Table 1:** Clinical characteristics

	Total (n = 1608)	HELT-E_2_S_2_ < 2 (n = 915)	HELT-E_2_S_2_ ≥ 2 (n = 693)	P value
Age, years	71.5 [64.0, 79.0]	66.0 [60.0, 71.0]	79.0 [75.0, 83.0]	< 0.001
Age 75–84 years, n (%)	503 (31.3%)	80 (8.7%)	423 (61.0%)	< 0.001
Age ≥ 85 years, n (%)	136 (8.5%)	0 (0%)	136 (19.6%)	< 0.001
Male, n (%)	1231 (76.6%)	771 (84.3%)	460 (66.4%)	< 0.001
Body mass index, kg/m^2^	23.6 [21.5, 25.7]	24.1 [22.2, 26.1]	22.8 [20.5, 25.1]	< 0.001
Body mass index < 18.5 kg/m^2^, n (%)	91 (5.7%)	11 (1.2%)	80 (11.5%)	< 0.001
Hypertension, n (%)	1201 (74.7%)	576 (63.0%)	625 (90.2%)	< 0.001
AF, n (%)	178 (11.1%)	12 (1.3%)	166 (24.0%)	< 0.001
Ischemic stroke, n (%)	155 (9.6%)	0 (0%)	155 (22.4%)	< 0.001
Diabetes, n (%)	594 (36.9%)	347 (37.9%)	247 (35.6%)	0.348
Dyslipidemia, n (%)	971 (60.5%)	575 (62.9%)	396 (57.2%)	0.021
Dialysis, n (%)	105 (6.5%)	46 (5.0%)	59 (8.5%)	0.005
Smoking, n (%)	908 (56.5%)	588 (64.3%)	320 (46.2%)	< 0.001
LEAD, n (%)	179 (11.1%)	58 (6.3%)	121 (17.5%)	< 0.001
Previous MI, n (%)	409 (25.4%)	235 (25.7%)	174 (25.1%)	0.793
Previous heart failure, n (%)	231 (14.4%)	86 (9.4%)	145 (21.0%)	< 0.001
Hemoglobin, g/dl	13.8 [12.6, 15.0]	14.2 [13.0, 15.3]	13.2 [12.0, 14.2]	< 0.001
eGFR, mL/min/1.73m^2^	62.4 [51.6, 75.0]	66.8 [55.0, 80.8]	56.6 [44.5, 65.2]	< 0.001
LVEF, %	62.9 [52.0, 69.0]	63.0 [52.0, 69.0]	62.0 [51.7, 69.3]	0.537
LVEF < 40%, n (%)	138 (9.0%)	75 (8.5%)	63 (9.6%)	0.468
CHADS_2_ score (range: 0–6)	2.0 [1.0, 2.0]	1.0 [1.0, 2.0]	3.0 [2.0, 3.0]	< 0.001
CHA_2_DS_2_-VASc score (range: 0–9)	3.0 [2.0, 4.0]	2.0 [1.0, 3.0]	4.0 [3.0, 5.0]	< 0.001
CREDO-Kyoto thrombotic risk score (range: 0–12)	1.0 [1.0, 3.0]	1.0 [0.0, 1.0]	3.0 [2.0, 4.0]	< 0.001
Clinical indication for PCI				
Chronic coronary syndrome, n (%)	925 (57.5%)	488 (53.3%)	437 (63.1%)	< 0.001
STEMI, n (%)	439 (27.3%)	270 (29.5%)	169 (24.4%)	0.022
NSTEMI, n (%)	87 (5.4%)	57 (6.2%)	30 (4.3%)	0.095
UAP, n (%)	157 (9.8%)	100 (10.9%)	57 (8.2%)	0.113
Medication at discharge				
Aspirin, n (%)	1538 (97.3%)	888 (98.2%)	650 (96.0%)	0.007
Thienopyridine, n (%)	1425 (90.1%)	839 (92.8%)	586 (86.6%)	< 0.001
OAC, n (%)	208 (12.9%)	75 (8.2%)	133 (19.2%)	< 0.001
Warfarin, n (%)	182 (11.3%)	69 (7.5%)	113 (16.3%)	< 0.001
DOAC, n (%)	26 (1.6%)	6 (0.7%)	20 (2.9%)	< 0.001
Statin, n (%)	1171 (74.2%)	727 (80.5%)	444 (65.8%)	< 0.001
ACE inhibitor or ARB, n (%)	1108 (68.9%)	625 (68.3%)	483 (69.7%)	0.551
β-blocker, n (%)	690 (43.9%)	400 (44.4%)	290 (43.2%)	0.624

Values are presented as n (%), or medians [interquartile range]

*AAA* abdominal aortic aneurysm, *ACE inhibitor* angiotensin-converting enzyme inhibitor, *AF* atrial fibrillation, *ARB* angiotensin receptor, *CK* creatine kinase, *DOAC* direct oral anticoagulant, *eGFR* estimated glomerular filtration rate, *HbA1c* hemoglobin A1c, *IABP* intra-aortic balloon pumping, *LEAD* lower extremity arterial disease, *LVEF* left ventricle ejection fraction, *MI* myocardial infarction, *NSTEMI* non-ST elevation myocardial infarction, *OAC* oral anticoagulant, *OMI* old myocardial infarction, *PCI* percutaneous coronary intervention, *STEMI* ST elevation myocardial infarction, *UAP* unstable angina pectoris

**Table 2:** Procedural characteristics

	Total (n = 1608)	HELT-E_2_S_2_ < 2 (n = 915)	HELT-E_2_S_2_ ≥ 2 (n = 693)	P value
Left main artery, n (%)	39 (2.4%)	18 (2.0%)	21 (3.0%)	0.171
Chronic total occlusion, n (%)	99 (6.2%)	59 (6.5%)	40 (5.8%)	0.773
Bifurcation, n (%)	453 (28.2%)	267 (29.2%)	186 (26.9%)	0.310
Multi-vessel disease, n (%)	616 (38.3%)	342 (37.4%)	274 (39.5%)	0.399
Number of stents	1.10 ± 0.739	1.07 ± 0.724	1.14 ± 0.758	0.062
Stent type				
New-generation DES	730 (45.4%)	394 (43.1%)	336 (48.5%)	0.030
Bare metal stent	605 (37.6%)	360 (39.3%)	245 (35.4%)	0.102
Total stent length, mm	22.0 [16.0, 30.0]	22.0 [16.0, 30.0]	22.0 [15.0, 32.0]	0.339
Mean stent diameter	3.0 [2.75, 3.5]	3.0 [3.0, 3.5]	3.0 [2.75, 3.5]	< 0.001

Values are presented as n (%), mean ± standard deviation, or median [interquartile range]. DES drug eluting stent

### Clinical outcomes

**Fig. 2:**
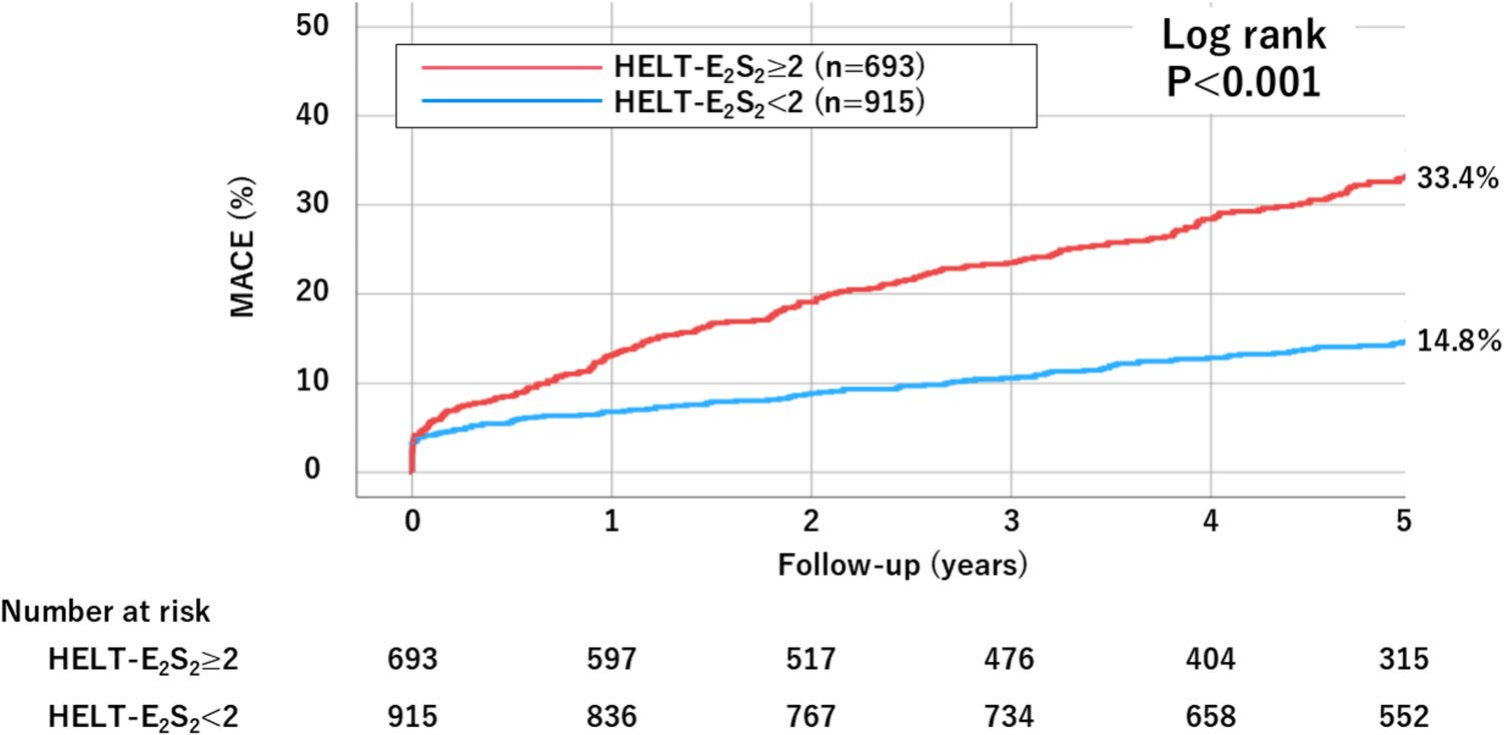
Kaplan–Meier curve for MACE at 5 years. *MACE* major adverse cardiovascular events

**Fig. 3:**
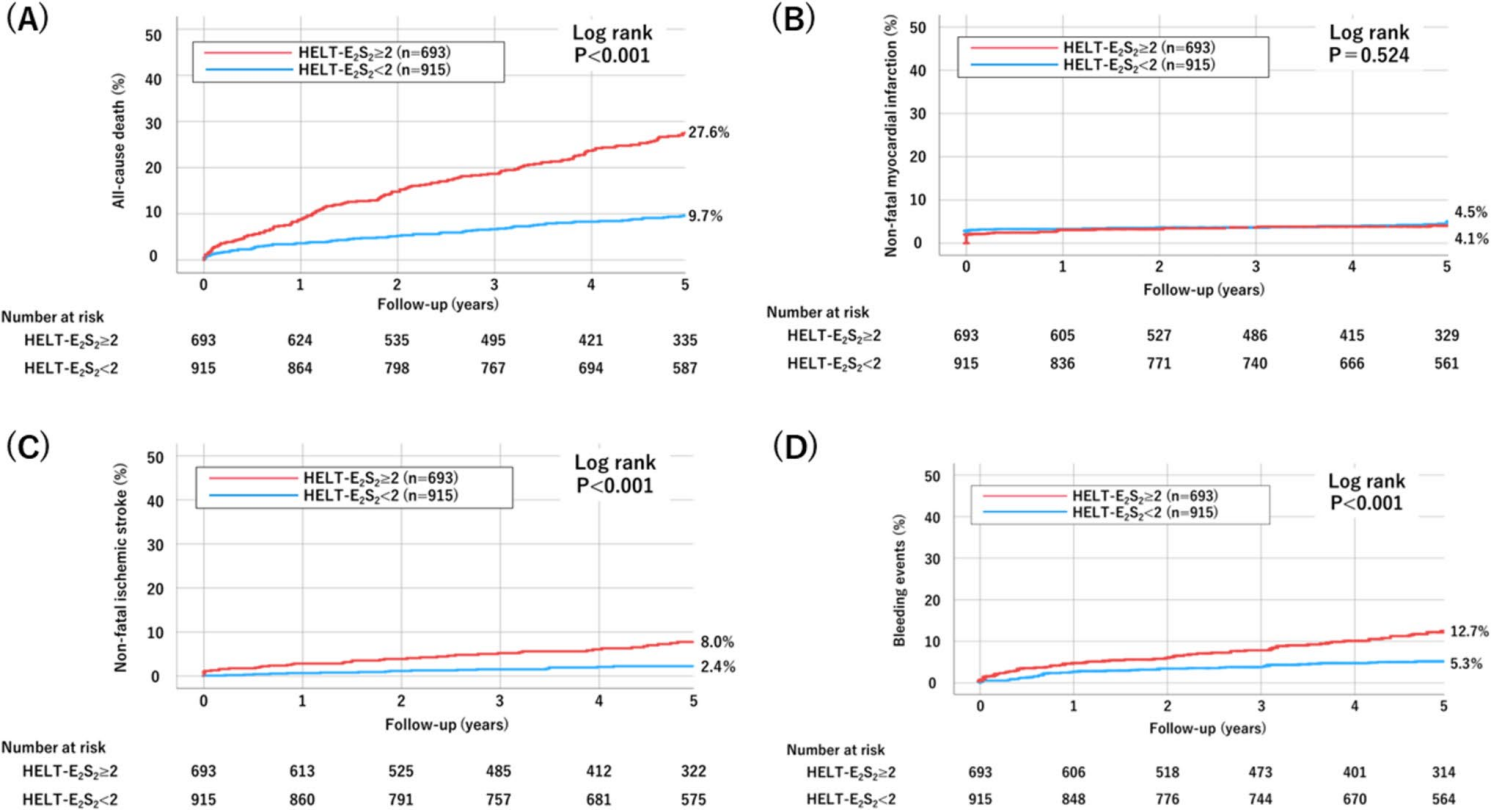
Kaplan–Meier curves for **A** all-cause death, **B** non-fatal myocardial infarction, **C** non-fatal ischemic stroke, and **D** BARC 2, 3 or 5 bleeding events. *BARC* Bleeding Academic Research Consortium

During a median follow-up period of 5 years (inter-quartile range: 3.4–5.0 years), there were 367 events of MACE (incidence rate: 5.22 per 100 patient-years), 280 all-cause deaths (incidence rate: 4.30 per 100 patient-years), 68 non-fatal MIs (incidence rate: 0.49 per 100 patient-years), 69 non-fatal strokes (incidence rate: 1.02 per 100 patient-years), 124 BARC 2, 3 or 5 bleeding events (incidence rate: 1.92 per 100 patient-years) (Table 3). The high HELT-E_2_S_2_ score group had an increased risk of MACE (33.4% vs. 14.8%, P < 0.001), all-cause death (27.6% vs. 9.7%, P < 0.001), non-fatal ischemic stroke (8.0% vs. 2.4%, P < 0.001), and BARC 2, 3 or 5 bleeding (12.7% vs. 5.3%, P < 0.001) (Figs. 2 and 3). There was no significant difference in myocardial infarction (4.1% vs. 4.5%, P = 0.524).

**Table 3:** Cox proportional hazards analysis of clinical outcomes by HELT-E_2_S_2_ score

	Total (n = 1608)	HELT-E_2_S_2_ < 2 (n = 915)	HELT-E_2_S_2_ ≥ 2 (n = 693) HR (95% CI) P value	Continuous HR (95% CI) P value
MACE	367 events	140 events	227 events	
Event rate (per 100 patient-years) (95%CI)	5.22 (4.68–5.82)	3.05 (2.54–3.67)	8.46 (7.40–9.69)	
Unadjusted		Reference	2.39 (1.93–2.95) P < 0.001	1.47 (1.37–1.59) P < 0.001
Adjusted		Reference	1.73 (1.11–2.69) P = 0.015	1.46 (1.25–1.72) P < 0.001
All-cause death	280 events	93 events	187 events	
Event rate (per 100 patient-years) (95%CI)	4.30 (3.82–4.83)	2.39 (1.95–2.92)	7.18 (6.22–8.29)	
Unadjusted		Reference	3.01 (2.34–3.85) P < 0.001	1.55 (1.43–1.69) P < 0.001
Adjusted		Reference	1.65 (1.01–2.72) P = 0.047	1.36 (1.13–1.64) P = 0.001
Non-fatal MI	68 events	42 events	26 events	
Event rate (per 100 patient-years) (95%CI)	0.49 (0.35–0.70)	0.45 (0.28–0.73)	0.55 (0.33–0.93)	
Unadjusted		Reference	0.85 (0.52–1.39) P = 0.527	1.11 (0.92–1.35) P = 0.281
Adjusted		Reference	1.75 (0.46–6.60) P = 0.410	1.68 (1.09–2.58) P = 0.018
Non-fatal stroke	69 events	20 events	49 events	
Event rate (per 100 patient-years) (95%CI)	1.02 (0.80–1.29)	0.49 (0.31–0.77)	1.81 (1.36–2.42)	
Unadjusted		Reference	3.71 (2.21–6.25) P < 0.001	1.60 (1.36–1.89) P < 0.001
Adjusted		Reference	4.67 (1.54–14.17) P = 0.006	2.00 (1.36–2.93) P < 0.001
BARC 2,3 or 5 bleeding events	124 events	49 events	75 events	
Event rate (per 100 patient-years) (95%CI)	1.92 (1.61–2.30)	1.29 (0.97–1.71)	2.88 (2.29–3.63)	
Unadjusted		Reference	2.39 (1.93–2.95) P < 0.001	1.47 (1.37–1.59) P < 0.001
Adjusted		Reference	1.45 (0.61–3.43) P = 0.399	1.20 (0.86–1.67) P = 0.298

Missing data: left ventricle ejection fraction (n = 73), hemoglobin (n = 2), estimated glomerular filtration rate (n = 55), chronic total occlusion (n = 5), bifurcation (n = 2). Of the study patients, 94.9% (1526/1608) were entered into the multivariable models. The HELT-E2S2 score was adjusted by the following covariates: for MACE and each component: male, dialysis, history of heart failure, diabetes, lower extremity artery disease, history of myocardial infarction, acute coronary syndrome, left ventricular ejection fraction less than 40%, hemoglobin, estimated glomerular filtration rate, and statin use at discharge; for BARC 2, 3 or 5 bleeding: male, dialysis, history of heart failure, lower extremity artery disease, acute coronary syndrome, hemoglobin, estimated glomerular filtration rate, oral anticoagulant use

*BARC* Bleeding Academic Research Consortium, *CI* confidence interval, *HR* hazard ratio, *MACE* major adverse cardiovascular events, *MI* myocardial infarction

As a sensitivity analysis, we divided patients into 4 groups based on HELT-E_2_S_2_ and CHADS_2_ score and assessed clinical outcomes. The HELT-E_2_S_2_ and CHADS_2_ scores were concordant in 77.6% of patients (HELT-E_2_S_2_ score ≥ 2 and CHADS_2_ score ≥ 2 [n = 641, 39.9%], HELT-E_2_S_2_ score < 2 and CHADS_2_ score < 2 [n = 607, 37.7%], HELT-E_2_S_2_ score ≥ 2 and CHADS_2_ score < 2 [n = 52, 3.2%], HELT-E_2_S_2_ score < 2 and CHADS_2_ score ≥ 2 [n = 308, 19.2%]). Kaplan–Meier analysis revealed the high HELT-E_2_S_2_ score and high CHADS_2_ score group had a higher incidence of MACE compared with other groups (Supplementary Fig. 2). Patients with discordance between the HELT-E_2_S_2_ and CHADS_2_ scores had an increased risk of MACE compared with those with low HELT-E_2_S_2_ score and low CHADS_2_ score.

### Cox regression analysis

The results of Cox proportional hazards analyses for clinical outcomes are summarized in Table 3. Multivariable Cox analysis demonstrated that, compared with the low HELT-E_2_S_2_ score (< 2), the high HELT-E_2_S_2_ score (≥ 2) was associated with an increased risk of MACE (HR, 1.73; 95% CI, 1.11–2.69, P = 0.015), all-cause death (HR, 1.65; 95% CI, 1.01–2.72, P = 0.047), and non-fatal stroke (HR, 4.67; 95% CI, 1.54–14.17, P = 0.006). Similarly, the HELT-E_2_S_2_ score as a continuous value (i.e. 0–7) emerged as an independent predictor for MACE, all-cause death, and non-fatal stroke (all, P < 0.05).

The C-statistics of the HELT-E_2_S_2_ score, CHADS₂ score, and CREDO-Kyoto thrombotic risk score for MACE at 5 years were 0.646, 0.642, and 0.696, respectively, and for ischemic stroke at 5 years were 0.667, 0.612, and 0.630, respectively.

## Discussion

This is the first study to demonstrate the prognostic value of HELT-E_2_S_2_ score for the long-term cardiovascular events among patients undergoing PCI. The major findings of the current study were (1) the high HELT-E_2_S_2_ score group had an increased risk of MACE, all-cause death, non-fatal ischemic stroke, and bleeding events in patients undergoing PCI, (2) the HELT-E_2_S_2_ score emerged as an independent predictor for MACE, while the prognostic value was similar between the HELT-E_2_S_2_ and CHADS₂ scores, and (3) there was a substantial discrepancy in the diagnosis of high-risk patients between HELT-E_2_S_2_ and CHADS₂ scores and patients with discordance had worse outcomes than those at low-risk defined by both scores.

An increased risk of MACE and all-cause death in the high HELT-E_2_S_2_ score group can be attributable to several factors. First, advanced age in the high HELT-E_2_S_2_ score group (i.e. ≥ 75 years old in 80% of patients) appeared to be a critical factor contributing to the increased risk of MACE and all-cause mortality. Elderly patients have more comorbidities and thus carry an increased risk of cardiac and non-cardiac death. Second, a higher incidence of stroke and bleeding events can also explain an increased risk of MACE and all-cause death. Advanced age is one of the established risk factors for stroke (i.e. CHADS_2_ score) and bleeding events (i.e. ARC-HBR criteria) [13]. Although data on the detailed medication status and a new occurrence of AF during the follow-up period were not available, a higher incidence of AF and anticoagulation might lead to a higher risk of stroke and bleeding. A close monitoring of new-onset of AF may be beneficial in patients with the high HELT-E_2_S_2_ score. Of note, the C-statistic of the HELT-E_2_S_2_ score for ischemic stroke was greater than that of CHADS_2_ score. In line with previous studies that consistently demonstrated a strong association between cardiovascular events and bleeding risks among patients undergoing PCI [14, 15], patients with the high HELT-E_2_S_2_ score also carried an increased risk of bleeding events in the current study. In this dilemmatic population, systematic application of the established bleeding risk score (e.g. ARC-HBR criteria and PRECISE-DAPT score) to determine adequate dual antiplatelet therapy (DAPT) duration and regimen appears clinically relevant to mitigate the ischemic as well as bleeding risks. Furthermore, to avoid unnecessary complex PCI (e.g. long or side branch stenting) may also be beneficial to shorten the required DAPT duration without increasing the risk of stent-related events. Our finding needs further investigation using the current dataset including patients treated with direct oral anticoagulant (DOAC). Interestingly, there was no significant difference in non-fatal MI between the high and low HELT-E_2_S_2_ score groups, despite more comorbidities in the high HELT-E_2_S_2_ score group. No significant difference in the PCI complexity may at least partly explain this comparable incidence of non-fatal MI.

Low BMI and extreme elderly, components of the HELT-E_2_S_2_ score, were common characteristics of Japanese patients with CAD [16–20]. Although these variables were not included in the CHADS_2_ and CHA_2_DS_2_-VASc scores, the discriminative ability of each score for MACE was similar in the current study. This can be explained by that the major components such as hypertension (74.7%), elderly (39.8%), and previous stroke (9.6%), were overlapping across the HELT-E_2_S_2_, CHADS_2_, and CHA_2_DS_2_-VASc scores. However, given the higher incidence of MACE in the high HELTE_2_S_2_ and low CHADS_2_ group (27.8%) than the low HELTE_2_S_2_ and high CHADS_2_ group (17.2%), categorical classification by the HELTE_2_S_2_ score (i.e. high or low risk) may have advantage for predicting future cardiovascular events over the CHADS_2_ score. The prognostic value of these scores should be investigated in other patient subsets such as LEAD and heart failure.

In the current study, CREDO-Kyoto thrombotic risk score, a validated risk score to identify Japanese patients with CAD at high risk for ischemic events [12], had the higher C-statistic for MACE than the HELTE_2_S_2_ score, while the C-statistic of the HELTE_2_S_2_ score for stroke was higher than that of CREDO-Kyoto thrombotic risk score. A previous study has reported that the C-statistic of CREDO-Kyoto thrombotic risk score for ischemic events (i.e. a composite of MI, definite or probable stent thrombosis, and ischemic stroke) were 0.680 in the derivation cohort and 0.640 in the validation cohort, respectively [12]. The better discriminative ability of CREDO-Kyoto thrombotic risk score for MACE was mainly due to the higher C-statistic for all-cause death (0.747 vs. 0.662). Given that the HELT-E_2_S_2_ score was originally developed to evaluate the risk of ischemic stroke in patients with AF, the HELT-E_2_S_2_ score may have an advantage to predict ischemic stroke compared with the previously-validated risk scores for ischemic events.

There are several limitations in the current study. First, this study was a post-hoc analysis of the prospective study. Second, although the persistent/permanent AF (not paroxysmal AF) was included as a component of the original HELT-E_2_S_2_ score, the type of AF was not available in the current study, which hindered to calculate the accurate HELT-E_2_S_2_ score. Third, sample size and number of events was relatively small. The lack of statistical significance between groups may be due to the lack of power (i.e. type II error). Fourth, there may be several potential unmeasured confounding factors inherent to observational data despite the efforts of multivariable adjustments. Finally, patients in the current study were enrolled between 2012 and 2013, and the management of patients undergoing PCI has significantly changed over the last decade. Limited use of new generation DES, transradial approach, statins, and DOAC in the current study cohort should be acknowledged when interpreting the findings of the current study. Furthermore, the target level of LDL cholesterol and the duration of antithrombotic therapy after PCI were also different from the current recommendation.

## Conclusion

The HELT-E_2_S_2_ score was strongly associated with an elevated risk for MACE, all-cause death, non-fatal ischemic stroke, and bleeding events in patients undergoing PCI. The HELT-E_2_S_2_ score appears a useful tool for predicting future cardiovascular events in CAD patients undergoing PCI.

## Supplementary Information

Below is the link to the electronic supplementary material.

Supplementary file1 (DOCX 212 KB)

## Data Availability

The data that support the findings of this study are available from the corresponding author upon reasonable request.
