# Supplementary material for: Prognostic value of HELT-E2S2 score among patients undergoing percutaneous coronary intervention: sub-analysis of the SHINANO 5-year registry
Source: Cardiovasc Interv Ther. 2025 Mar 29;40(3):544–52. doi: 10.1007/s12928-025-01123-8 (PMC12167246; doi:10.1007/s12928-025-01123-8)
Supplement: Supplementary file 1 — Supplementary file1 (DOCX 212 KB) [file 12928_2025_1123_MOESM1_ESM.docx]

**Supplementary Figure 1.** Overlap of HELT-E_2_S_2_ score.

**
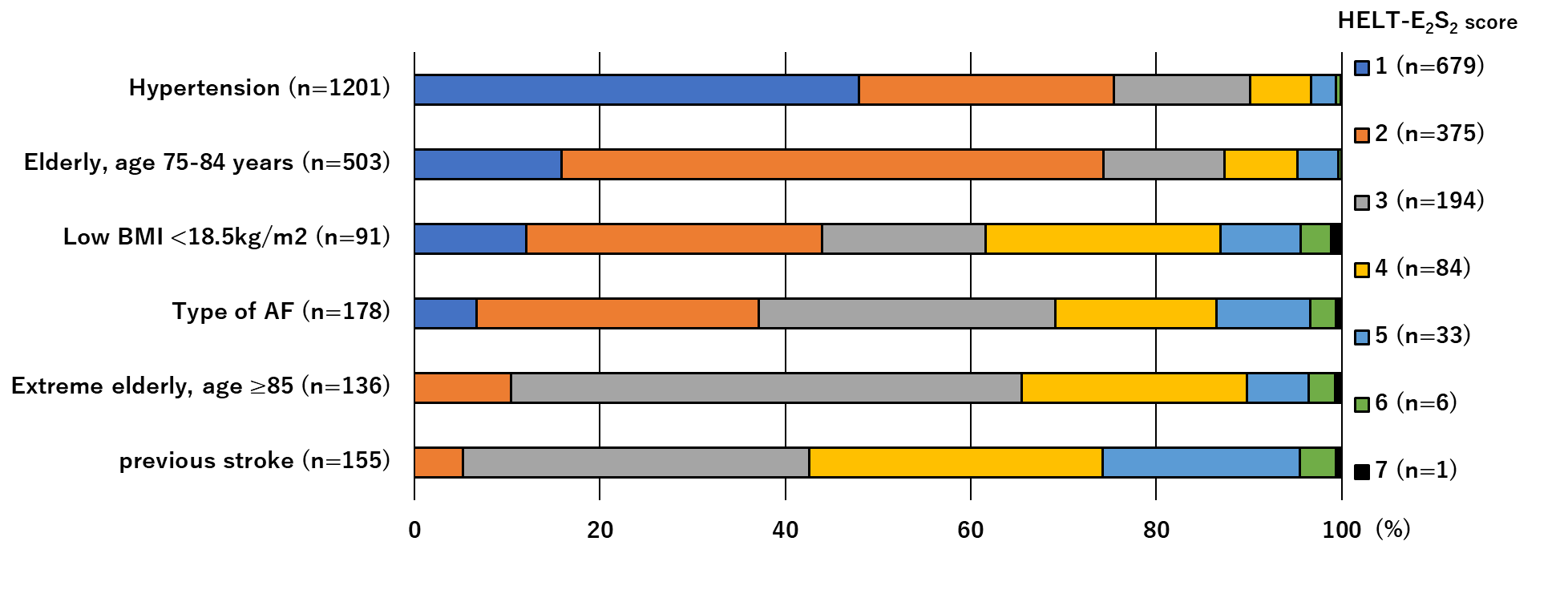
**

AF, atrial fibrillation; BMI, body mass index

**Supplementary Figure 2.** Kaplan-Meier curves for MACE at 5 years according to the HELT-E_2_S_2_ and CHADS_2_ scores.


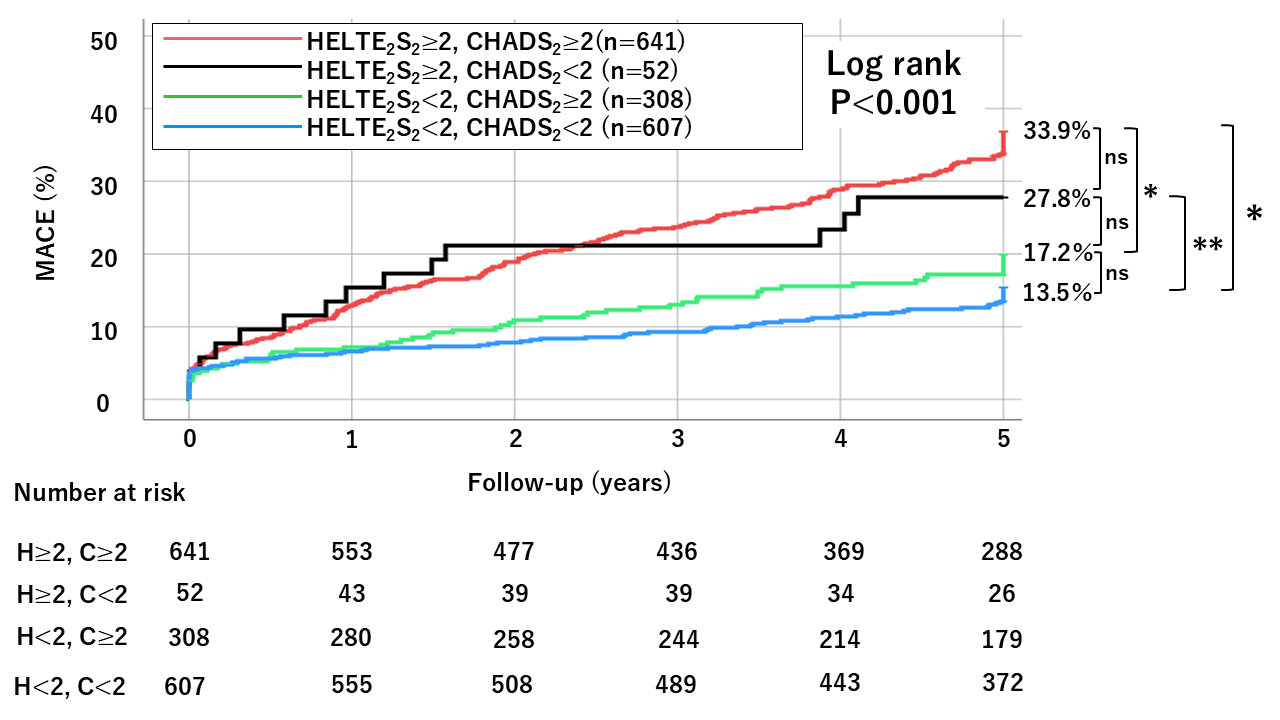


* indicates P <0.001, and ** indicates P<0.05.

MACE, major adverse cardiovascular events; ns, not significant.
